# Comparative Evaluation of Rapid Nucleic Acids Extraction Methods for Biosensor-Based Point-of-Care Solutions

**DOI:** 10.3390/bios16040195

**Published:** 2026-03-28

**Authors:** Maciej Polak, Aldona Wiatrzyk, Katarzyna Krysztopa-Grzybowska, Karolina Sobiecka, Ewa Mosiej, Marta Prygiel, Robert Ziółkowski, Dawid Jańczak, Katarzyna Pancer, Aleksandra Skiba, Aleksandra Anna Zasada

**Affiliations:** 1 Department of Sera and Vaccines Evaluation, National Institute of Public Health NIH—National Research Institute, 00-791 Warsaw, Poland; mpolak@pzh.gov.pl (M.P.); awiatrzyk@pzh.gov.pl (A.W.); kkrysztopa@pzh.gov.pl (K.K.-G.); ksobiecka@pzh.gov.pl (K.S.); emosiej@pzh.gov.pl (E.M.); mprygiel@pzh.gov.pl (M.P.); 2 Chair of Medical Biotechnology, Faculty of Chemistry, Warsaw University of Technology, 00-664 Warsaw, Poland; robert.ziolkowski@pw.edu.pl (R.Z.); aleksandra.skiba@pw.edu.pl (A.S.); 3 Department of Infectious and Invasive Diseases and Veterinary Administration, Institute of Veterinary Medicine, Faculty of Biological and Veterinary Sciences, Nicolaus Copernicus University, 87-100 Toruń, Poland; djanczak@umk.pl; 4 Animallab Veterinary Laboratory, 02-672 Warsaw, Poland; 5 Department of Virology, National Institute of Public Health NIH—National Research Institute, 00-791 Warsaw, Poland; kpancer@pzh.gov.pl

**Keywords:** rapid point-of-care (POC) diagnostic tests, nucleic acid amplification (NAA) methods, recombinase polymerase amplification (RPA)

## Abstract

The translation of nucleic acid amplification into practical point-of-care and biosensor-integrated diagnostics is still significantly impeded by the necessity for rapid sample preparation. For this reason, a broad comparison of seven commercially available kits for DNA/RNA extraction containing their temperature-related adjustments was performed. Extracts isolated from SARS-CoV-2-positive nasopharyngeal swabs, viral stocks, as well as laboratory-prepared suspensions of clinically relevant Gram-positive and Gram-negative bacteria were evaluated by recombinase polymerase amplification (RPA) and real-time PCR. In addition, the impact of transport media for SARS-CoV-2 samples was investigated. Extraction performance varied markedly according to the kit, pathogen, sample background. For SARS-CoV-2, rapid extraction was more effective for samples collected in viral transport medium than in inactivation buffer. Across bacterial targets, performance was species dependent, highlighting substantial differences in compatibility between simplified extraction workflows and downstream amplification. Among the rapid methods tested, a simplified QuickExtract protocol (95 °C, 5 min) provided the most consistent overall results, although it did not uniformly match the reference silica-based method for all targets. In conclusion, these results demonstrate that rapid nucleic acid extraction must be thoroughly evaluated as an essential element of the entire sample-to-answer workflow, rather than being chosen as a standalone preprocessing step for point-of-care molecular diagnostics.

## 1. Introduction

Development of point-of-care (POC) diagnostic medical devices has been a strong trend in recent years. A special need for rapid POC testing has been identified for antimicrobial susceptibility testing and pathogen identification as a response to global public health threats [1]. Biosensors are a crucial technology applied for POC testing development [2]. Among them, DNA-based biosensors play an important role [3].

Nucleic acid extraction is a critical step in all RNA/DNA-based methods. Usually, many manual steps and additional instruments are required for the preparation of DNA/RNA samples. The most frequently used nucleic acid extraction methods include classic phenol-chloroform extraction, solid phase extraction where the solid phase is created from silica beads, sol-gels or ion exchange resins, and modern magnetic beads extraction. The methods require several centrifugation steps or negative pressure (vacuum) to drive the sample and buffers through the solid phase (loading, washing, and elution), or a magnet to capture the magnetic beads. In addition, numerous pipetting stages are necessary. Therefore, the methods are not suitable for rapid POC testing.

The aim of any nucleic acid extraction method is to obtain DNA/RNA of purity appropriate for analytical methods used for sample analysis, e.g., amplification, hybridisation, sequencing, etc. Nucleic acid amplification methods, such as PCR and various isothermal amplification methods, are the most broadly applied methods in molecular diagnostics of infections as well as detection and identification of microorganisms in clinical samples. Recently, rapid DNA/RNA extraction kits have appeared on the market mainly as a response to needs related to the COVID-19 pandemic. In the study, the authors compared selected commercially available kits for DNA/RNA extraction in terms of their suitability for POC tests based on DNA/RNA amplification.

## 2. Materials and Methods

### 2.1. Clinical and Laboratory-Prepared Samples

Thirty nasopharyngeal swab specimens were collected from patients with suspected COVID-19 in 2021. The presence of SARS-CoV-2 in the specimens was confirmed by routine laboratory diagnostic tests as described previously [4]. The study was approved by the Bioethics Committee of the National Institute of Public Health—the National Institute of Hygiene (NIPH-NIH). Each patient gave written consent to participate in the study. The swabs were transported in the CITOSWAB viral transport medium (VTM) (CITOTEST Scientific, Nanjing, China) or inactivation buffer (Virus RNA Collection Kit, Zeesan, Xiamen, China).

In addition, viral and bacterial laboratory-prepared samples were used. The SARS-CoV-2 cultures were obtained from two clinical specimens using the Vero E6 cell line. The initial yield of each viral stock, assessed as previously described, was 10^6^ per mL. The stocks were inactivated for 15 min at 65 °C. The bacterial species for the study were selected from the WHO Bacterial Priority Pathogen List, 2024 [1] and included the following: *Escherichia coli* ATCC 25922, *Klebsiella pneumoniae* ATCC 700603 (WHO critical group), *Shigella dysenteriae* 17, *Enterococcus faecium* DSM20477T, *Salmonella enterica* subsp. *enterica* serovar Typhimurium DSM19587, *Staphylococcus aureus* DSM20231T, *Pseudomonas aeruginosa* ATCC 27853 (WHO high group), and *Streptococcus pneumoniae* PCM2589 (WHO medium group). Additionally, *Corynebacterium diphtheriae* NCTC13129 and *Clostridium perfringens* 1064 strains were used. Bacteria were cultured aerobically or anaerobically (GENbag anaer, Biomerieux, Marcy-l’Étoile, France) at 37 °C, initially on Agar medium (Tryptic Soy Agar, Brain Heart Infusion Agar, or Columbia Agar with 5% sheep blood) and then twice on liquid medium (Tryptic Soy Broth or Brain Heart Infusion Broth). The final passage was prepared by inoculating fresh medium with an overnight culture of bacteria at a ratio of 1:100. Propagation continued until mid-logarithmic growth was achieved. Bacterial cell numbers were determined by spreading their ten-fold serial dilutions (50–100 µL) onto the agar media. Ten-fold serial dilutions of prepared virus stocks and the bacterial cultures in PBS (with peptone addition for bacteria), were also used to determine the limit of detection (LOD) of the real-time PCR method.

### 2.2. DNA/RNA Extraction

In this study, following kits and buffers for rapid nucleic acids extraction dedicated to PCR and isothermal amplification were used: ViRNAEx (MetaCell, Praha, Czech Republic), QuickExtract (LGC, Guildford, UK), One-Step DNA/RNA Extraction Buffer (CHAI, Santa Clara, CA, USA), Loopamp PURE DNA Extraction Kit (EIKEN Chemical, Tokyo, Japan) for all types of samples and Loopamp Viral RNA Extraction Kit (EIKEN Chemical, Tokyo, Japan) for viral samples only. DNeasy Blood and Tissue Kit (QIAGEN, Hilden, Germany) and QIAamp Viral RNA Mini Kit (QIAGEN, Germany), which are silica-based methods, were used as references. The extraction was conducted according to manufacturer’s instructions. Additionally, several modifications of the kits’ procedures were tested. Each kit was tested with and without addition of carrier RNA (QIAGEN, Germany) in the case of samples containing SARS-CoV-2. For viral samples, the extraction with QuickExtract was conducted in modified incubation time and temperature: 10 min at 65 °C and from 1 to 5 min at 92 °C, 95 °C, 98 °C. For bacterial samples, the following one-step modifications of the QuickEtract procedure were tested: 10 min at 65 °C and 5 min at 95 °C.

The volume of samples collected for DNA and RNA isolation was 50 µL. Liquid media without target organisms were used as a negative extraction control.

### 2.3. Real-Time PCR

The extracted samples were subjected to real-time PCR. For clinical and laboratory-prepared SARS-CoV-2 samples the AmoyDx Novel Coronavirus (2019-nCoV) Detection Kit (AmoyDx, Singapore) was used, which combines reverse transcription and PCR amplification of ORF1ab and gene N markers in one-step procedure (real-time RT-PCR). The volume of template was 2 µL and 9 µL. The reaction was conducted according to manufacturer’s instruction, using the CFX Opus 96 (Bio-Rad, Hercules CA, USA) apparatus.

For bacterial samples, real-time PCR was performed in 20 μL reactions containing 200 nM of each primer (Table 1), 10 μL 2 × SsoAdvanced Universal SYBR Green Supermix (Bio-Rad, Hercules CA, USA), and 1 µL extracted DNA. CFX Opus 96 Real-Time System (Bio-Rad, Hercules CA, USA) apparatus was used for thermal cycling: initial denaturation at 98 °C for 3 min, followed by 40 cycles of denaturation at 98 °C for 15 s and annealing at 65 °C for 40 s. Results were analysed using the CFX Manager Software version 2.3 (Bio-Rad, USA).

Both assays included a positive control (SARS-CoV-2 target gene template or purified bacterial DNA), a non-template control, and a negative extraction isolation control.

### 2.4. Isothermal Amplification

The extracted DNA/RNA samples were subjected to Recombinase Polymerase Amplification (RPA) method using TwistAmp Basic Kit (TwistDx, Maidenhead, UK), according to the manufacturer’s instruction. For SARS-CoV-2 clinical specimens two markers were amplified: RdRP and gene E. Primers used for RdRP amplification were as follows: nCoV_IP4-Fw: GGTAACTGGTATGATTTCG and nCoV_IP4-Rv: CTGGTCAAGGTTAATATAGG. For gene E amplification, we used primers described by Corman et al. [15]. For bacterial samples, we used the same primers as for real-time PCR (Table 1). After the reaction, the samples were mixed with 6X DNA Loading Dye & SDS Solution (ThermoScientific, Waltham MA, USA) and heated at 65 °C for 10 min. Amounts of 2.5 μl of the RPA product were loaded on 2% agarose gel. The presence and size of the amplicons stained by GelRed (Biotium, Fremont CA, USA) were verified by comparison with the molecular DNA marker Perfect 100 bp Ladder (EURx, Poland). The study included two positive controls (control included with the test and purified target DNA/RNA template), a non-template control, and a negative extraction control.

### 2.5. Statistical Analysis

The statistical differences between the various DNA/RNA extraction methods were compared using the real-time PCR quantification cycle (Cq) values of each sample. The Shapiro-Wilk test was used to verify whether the sample data distribution was close to a normal distribution. Based on that, the paired *t*-test, Mann–Whitney U-test and ANOVA were used in further analysis. *p*-value ≤ 0.05 was considered as statistically significant.

## 3. Results

### 3.1. Extraction of Viral RNA

To evaluate the presence of amplifiable RNA and assess PCR inhibitors in the samples, the authors applied real-time RT-PCR targeting two SARS-CoV-2 markers: ORF1ab and N gene. In the first stage of the study, we used six nasopharyngeal swabs taken from COVID-19-positive patients: three swabs transported in inactivation buffer and three swabs in VTM. We obtained positive real-time RT-PCR results for five out of six (at least in one marker) clinical samples extracted with reference silica-based method, regardless of the template volume used for the reaction (2 or 9 µL). Cq values of the samples were between 21.66 and 37.09. For samples extracted using ViRNAEx and QuickExtract we obtained positive results only for those transported in VTM (2/3 positive swabs) but not in the inactivation buffer. Moreover, for ViRNAEx-extracted samples, PCR was positive mostly when 2 µL of template was used. Cq values were comparable to those calculated for silica-based extraction. We did not obtain real-time RT-PCR positive results when the RNA was extracted using the Loopamp Viral RNA Extraction Kit, Loopamp PURE DNA Extraction Kit and One-Step DNA/RNA Extraction Buffer, regardless of the template volume. The use of Carrier RNA during extraction did not impact the results for all the extraction kits.

**Figure 1:**
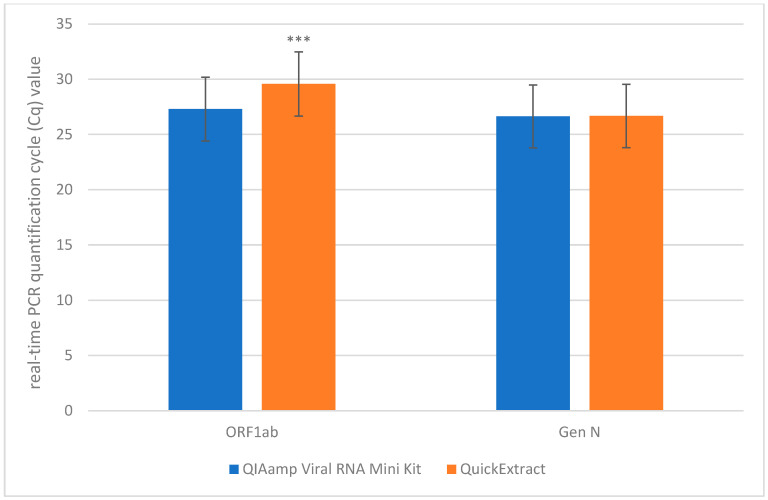
Comparison of mean Cq values obtained for SARS-CoV-2 clinical samples extracted with silica-based reference method (QIAamp Viral RNA Mini Kit) and rapid method using QuickExtract (95 °C, 5 min). *** *p*-value < 0.001.

Based on the above results, we selected QuickExtract for further investigation. To simplify the QuickExtract protocol, which originally included two incubation steps (6 min at 65 °C followed by 2 min at 98 °C), we applied one-step incubation: 10 min at 65 °C and from 1 to 5 min at 92 °C, 95 °C, 98 °C. The results obtained for three clinical specimens are presented in Table 2. We did not observe significant differences in Cq value between samples incubated for 1, 2, 3 and 5 min (*p*-value 0.5423 for ORF1ab; *p*-value 0.9920 for gene N) as well as between samples incubated at 92 °C, 95 °C and 98 °C (*p*-value 0.4448 for ORF1ab; *p*-value 0.9981 for gene N). Therefore, in further analysis using a larger number of clinical samples (15 positive, 3 negative and 3 inconclusive according to routine diagnostic test results), we used a simplified one-step extraction protocol with incubation for 5 min at 95 °C. Cq values for gene N marker were comparable between samples extracted with QuickExtract one-step protocol than with QIAamp Viral RNA Mini Kit (average difference: 0.04; median: 0.02). The obtained difference in Cq values for ORF1ab marker indicated remarkably high statistical significance between samples extracted with these methods (*t*-value 10.7773, *p*-value 3.6676 × 10^−8^) (Figure 1.). The average difference for OFR1ab marker was 0.63 (median: 0.93).

In the next step of the study, we used 10-fold dilutions (ranging from 10^−2^ to 10^−7^) of two laboratory-prepared SARS-CoV-2 cultures (10^6^ particles per ml). In samples isolated with both QuickExtract and QIAamp Viral RNA Mini Kit methods, the SARS-CoV-2 ORF1ab and N gene targets were detected at dilutions of up to 10^−5^ (100 viral particles per mL). Additionally, in one case, a positive ORF1ab result was observed in QIAamp Viral RNA Mini Kit isolated sample at a dilution of 10^−6^ (10 viral particles per ml). As with the clinical samples, no positive amplification results were obtained for either marker across all dilutions of laboratory-prepared samples isolated using Loopamp Viral RNA Extraction Kit.

All the viral clinical samples that were real-time RT-PCR-positive were also positive when analysed using the RPA method.

### 3.2. Extraction of Bacterial DNA

**Figure 2:**
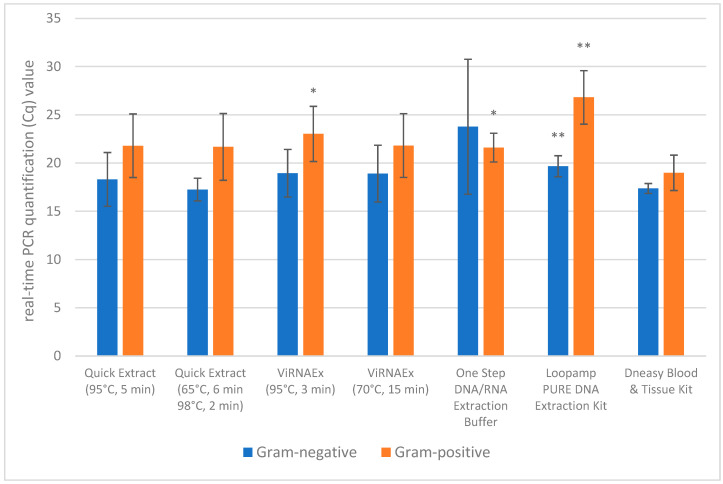
Comparison of mean Cq values obtained for bacterial DNA samples extracted with various methods. * *p* < 0.05; ** *p* < 0.01.

To evaluate the presence of amplifiable DNA in the samples, the authors applied real-time PCR and RPA methods. In the first experiment, we compared results obtained for DNA extracted from pure bacterial cultures harvested in mid-log phase (containing approximately 10^5^ cfu/µL). For quantitative real-time PCR experiments, two biological and three technical replicates were performed, while for the end-point RPA experiment, a single replicate was performed. For Gram-negative bacteria, Cq values of the samples extracted with DNeasy Blood & Tissue Kit were between 16.05 and 18.84 (median 17.26), and for Gram-positive, between 15.66 and 24.93 (median 17.96). Except for with the One Step DNA/RNA Extraction Buffer method, the Cq values obtained for samples extracted from Gram-negative bacteria were lower than those from Gram-positive ones. More statistically significant differences in Cq values obtained with the rapid methods compared to the reference method were observed for Gram-positive bacteria. For this group of interest, three out of six methods—ViRNAEx (95 °C, 3 min), One-Step DNA/RNA Extraction Buffer and Loopamp PURE DNA Extraction Kit—showed significantly higher Cq values than those extracted with the reference method (ViRNAEx: t-value 3.1668, *p*-value 0.0340, One-Step DNA/RNA Extraction Buffer: *t*-value 4.4803, *p*-value 0.0110, Loopamp PURE DNA Extraction Kit: *t*-value 6.7900, *p*-value 0.0025). A significant difference for Gram-negative bacteria samples was observed only for Loopamp PURE DNA Extraction Kit method (*t*-value 5.4568, *p*-value 0.0055). The smallest differences in Cq in both sample groups in comparison with reference method were observed for QuickExtract methods (two-step: 65—6 min, 98—2 min and one-step: 95—5 min). The efficiency of DNA extraction with QuickExtract for 10 min at 65 °C was highly dependent on bacterial species. We did not obtain real-time PCR positive results for *S. pneumoniae*, *C. perfringens* and *C. diphtheriae*, and the results for *K. pneumoniae*, *S. dysenteriae*, *S.* Typhimurium and *E. feacium* were not conclusive due to poor repeatability of the results. We obtained positive results only for *E. coli*, *P. aeruginosa* and *S. aureus* with Cq values comparable to the reference method (*p*-value 0.5245). Comparison of ViRNAEx methods (95—3 min and 70—15 min) as well as QuickExtract methods (one-step: 95—5 min and two-step: 65—6 min, 98—2 min) indicated lack of their significant difference (respectively, *p*-values for Gram-negative bacteria: 0.8948 and 0.3596 as well as *p*-values for Gram-positive bacteria: 0.0620 and 0.5581). Considering all bacterial species analysed in this study, the QuickExtract kit was found to be the most comprehensive of the rapid extraction methods tested compared to the reference method. The comparison of investigated extraction methods is shown in Figure 2.

A comparison of extraction kits in terms of time consumption, complexity of execution and required equipment is presented in Table 3.

**Figure 3:**
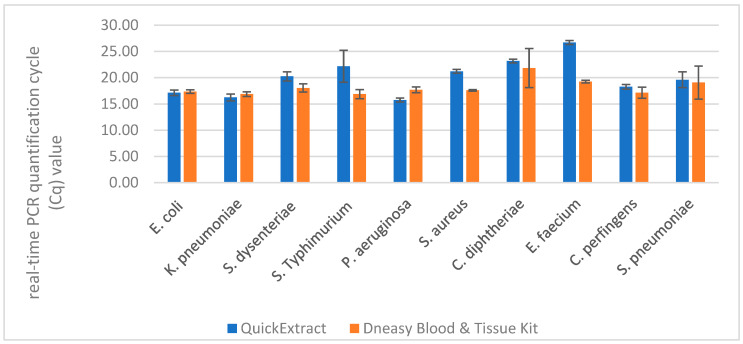
Comparison of mean Cq values obtained for DNA samples extracted with QuickExtract (95 °C, 5 min) and DNeasy Blood & Tissue Kit (reference method) from various bacterial species.

We also noticed that species of bacteria was an important factor in the efficiency of DNA extraction. Even for the reference method, we observed differences in Cq values, for example the calculated Cq value difference between *K. pneumoniae* and *C. diphtheriae* amounted to 4.97. The comparison of mean Cq values obtained for each species after DNA extraction with QuickExtract (95 °C, 5 min) and DNeasy Blood & Tissue Kit are presented in Figure 3.

RPA results for most bacteria were consistent with those obtained by real-time PCR. Positive isothermal amplification results were obtained for all tested species, whose DNA was isolated using the following methods: QuickExtract (two-step: 65—6 min, 98—2 min and one-step: 95—5 min), ViRNAEx (95—3 min and 70—15 min), Loopamp PURE DNA Extraction Kit and DNeasy Blood & Tissue Kit. For *E. faecium*, RPA products were present in all DNA isolation methods. Additionally, similarly to real-time PCR experiments, negative RPA results were obtained for DNA samples of 8 species isolated using QuickExtract method with a reduced incubation temperature (10 min at 65 °C). However, unlike real-time PCR, no positive RPA results were obtained for *S.* Typhimurium and *K. pneumoniae* samples isolated using One-Step DNA/RNA Extraction Buffer.

In the next step of the study, bacterial culture dilutions (from 10^−2^ to 10^−7^) for the extraction with Loopamp PURE DNA Extraction Kit, QuickExtract and DNeasy Blood & Tissue Kit were used. At each dilution level, three biological and four technical replicates were performed with real-time PCR. The limit of detection, indicated as the highest dilution with consistent amplification in ≥90% of replicates, in cfu/µL, for each bacterial species together with the obtained mean Cq value is presented in Table 4. We observed comparable LOD for Gram-positive and Gram-negative bacteria extracted with DNeasy Blood & Tissue Kit (*p*-value: 0.6761) and significantly higher values for QuickExtract and Loopamp PURE DNA Extraction Kit (*p*-value of 0.0122 and 0.0216, respectively). Comparison of the rapid DNA isolation methods with the reference method showed no statistically significant differences for Gram-negative bacteria (QuickExtract *p*-value: 0.6723, Loopamp PURE DNA Extraction Kit *p*-value: 0.2462) and significant ones for Gram-positive bacteria (QuickExtract *p*-value: 0.0465 Loopamp PURE DNA Extraction Kit: 0.0367).

## 4. Discussion

Nucleic acid extraction is the most crucial step in any molecular biology technique. The key element of development of a successful DNA/RNA extraction protocol is the application of standardised reagents. As commercially available kits are subject to quality control, we decided to test selected kits on a wide range of samples to compare their performance against reference methods. Some modifications to the QuickExtract protocol were tested to simplify it as much as possible and adapt to POC applications.

Successful extraction of high-quality nucleic acids from biological samples requires sufficient disruption of the cells, denaturation of nucleoprotein complexes and inactivation of cell nucleases, as well as final purification of nucleic acids. These multi-step processes need to be simplified for rapid tests and POC devices. Cell wall composition is an important factor impacting the effectiveness of cell disruption, nucleic acid extraction efficiency and downstream amplification. For example, Gram-positive bacteria have teichoic acids, a thicker peptidoglycan layer and more rigid cell wall than Gram-negative bacteria. Therefore, in the case of Gram-positive bacteria, the extraction process requires the use of more aggressive reagents, such as lysozyme, lysostaphin, dithiothreitol (DDT), concentrated alkaline buffers, guanidine, Triton X-100, sodium dodecyl sulphate (SDS), etc. [16,17,18]. Additionally, some bacteria, such as *Mycobacterium* sp. and *Corynebacterium* sp., contain mycolic acids in cell wall structures, which make them even more difficult to disrupt [19].

Gram-negative bacteria contain lipopolysaccharides (LPS), which can co-purify with nucleic acids and interfere with polymerase activity [20,21]. In addition, polysaccharide capsules and extracellular polymeric substances (EPS) may further reduce amplification efficiency by increasing viscosity or interacting with enzymatic reactions [21,22].

A great challenge is DNA extraction from spores, such as *Bacillus anthracis* spores, which are highly resistant to many chemical and physical factors [23]. There are also differences between enveloped and non-enveloped viruses, as in non-enveloped viruses the nucleic acids are protected by a protein capsid which has to be disrupted during the extraction process [24]. Moreover, in the case of clinical samples and intercellular pathogens, the eucaryotic cells need to be disrupted to reach the pathogen DNA/RNA. The results of our study confirmed the significant influence of bacterial cell wall structure on the efficiency of DNA extraction with rapid extraction kits.

Another important step in the DNA/RNA extraction procedure is the removal of inhibitory substances that might be present in a sample. Inhibitory substances have a negative impact on nucleic acid amplification by interfering with polymerases, degrading or capturing nucleic acids or interfering with the extraction of nucleic acids [25]. Different inhibitors might be present in various clinical samples, for example haemoglobin, lactoferrin, collagen, melanin, hematin and heparin in blood and blood-derived samples (plasma, serum, etc.), bile salts, polysaccharides, urea in stool and urine samples, and even medicines such as antiviral substances (e.g., acyclovir), etc. [20,26]. Improvement of inhibitor-tolerant polymerases, as well as isothermal DNA amplification methods, which are more tolerant to inhibitors than conventional PCR, have had a strong impact on the development of rapid nucleic acid amplification (NAA) tests and POC devices [27,28,29]. The application of POC rapid tests also reduces the nucleic acid degradation due to a prompt procedure directly after sampling. Thus, the template for the reaction does not need to be highly purified.

Importantly, our study provides practical insights into the influence of inhibitors on rapid extraction protocols. Clinical nasopharyngeal samples contain various endogenous (e.g., mucins, host proteins, enzymes, and blood components) and exogenous (e.g., intranasal medications and components of transport media) inhibitors that can interfere with nucleic acid extraction and amplification [26,30,31].

Endogenous inhibitors may increase sample viscosity and impair polymerase activity, while exogenous substances can further reduce amplification efficiency. These inhibitors are particularly relevant in simplified extraction protocols that do not include extensive purification steps, as their presence may lead to increased Cq values or false-negative results [26,32]. Recent studies also emphasize that complex biological matrices frequently contain compounds that inhibit nucleic acid amplification and complicate downstream molecular detection, highlighting the need for robust extraction strategies compatible with rapid diagnostic workflows [33]. We demonstrated that inhibitors present in nasopharyngeal samples do not influence on amplification reaction when nucleic acids are extracted with QuickExtract but inhibit the reaction when extracted with ViRNAEx. The negative results of amplification obtained for samples extracted with the Loopamp Viral RNA Extraction Kit, Loopamp PURE DNA Extraction Kit and One-Step DNA/RNA Extraction Buffer might be related to the presence of inhibitors or to the insufficient RNA release from virus particles and epithelial cells.

Comparison of five rapid nucleic acid extraction systems under various incubation conditions shows that the optimised QuickExtract protocol—a single 5 min incubation at 95 °C—reliably releases nucleic acids from clinical viral and diverse bacterial samples. In addition, this one-step protocol supports robust amplification using both PCR and isothermal methods such as recombinase polymerase amplification (RPA), which are known to tolerate residual inhibitors in complex biological matrices [34]. Results obtained from pure bacterial cultures confirmed that differences in amplification were primarily attributable to the intrinsic efficiency of the extraction methods rather than matrix effects.

Silva et al. [21] demonstrated the usefulness of two rapid nucleic acid extraction methods, Pi-Lise and QuickExtract, for SARS-CoV-2 detection in clinical samples with high viral load and low viral load transported in VTM and saline. We confirmed their findings for QuickExtract and extended the study by including additional rapid nucleic acid extraction methods and wider range of samples (SARS-CoV-2 stocks with known virus yield, additional transport media for COVID-19 diagnostic samples and various Gram-positive and Gram-negative bacterial pathogens). We revealed that some transport media in commercial sampling kits designed for virology diagnostics might negatively influence the nucleic acid extraction efficiency. For example, when it comes to NAA, we did not obtain positive amplification results for viral samples transported in inactivation buffer. Our study also showed that smaller volume of extracted RNA/DNA used for an amplification reaction might result in better amplification, as we observed for samples extracted with ViRNAEx. Sample dilution may reduce inhibitor concentration, but it may also impact assay sensitivity by reducing the number of target RNA/DNA copies. Adding poly-A carrier RNA to samples during the extraction process may enhance RNA/DNA recovery [22]. However, we did not observe any effect of poly-A carrier RNA on amplification efficiency.

The study of Alemán-Duarte et al. [35], based on a multi-step cetyltrimethylammonium bromide (CTAB)-double-phenol extraction method, reported that DNA yield was significantly lower in Gram-positive bacteria than in Gram-negative. We found no statistically significant differences between silica-based DNeasy Blood & Tissue Kit results for these two bacterial groups, but such differences were observed for the Loopamp PURE DNA Extraction and QuickExtract methods. Similarly to the authors, in our study, differences in DNA extraction efficiency between bacterial species were reflected in the Cq values obtained in real-time PCR.

The most common extraction methods used for rapid NAA tests are heating in distilled water, saline or PBS, NaOH treatment and a magnetic beads method (examples: [36,37,38]). However, these methods have some disadvantages. For example, heating can lead to denaturation of proteins and enzymes inactivation but may also degrade nucleic acids [39,40]. Moreover, some bacterial pathogens produce heat-resistant proteases that may not be disrupted by heating and cause NAA inhibition [41]. The heat shock method applied for RNA extraction from nasopharyngeal swab samples with SARS-CoV-2 resulted in a very wide Cq difference (10 Cq) in comparison to extraction with a commercial kit. Moreover, samples with low viral load (Cq above 30) were false negative in the heat shock method [39]. NaOH treatment leads to cell lysis and, in consequence, nucleic acid release. However, the high pH may degrade DNA. Also, alkaline conditions may denature proteins, including enzymes and proteins involved in DNA amplification, and cause NAA inhibition [39]. Chaudhary et al. [42] compared four DNA extraction methods: silica membrane-based spin column method, magnetic bead method, paper-based dipstick method and heat NaOH treatment method (HotSHOT). The study revealed that in the HotSHOT method, the extracted DNA was fragmented and impure, thus failing in PCR, but yielded strong loop-mediated isothermal amplification (LAMP). This is because enzymes used for amplification in isothermal conditions are more resistant to inhibitors than Taq polymerase. Also, Liu et al. [36] presented that NaOH treatment of clinical samples, such as blood and saliva, for DNA extraction caused the mixture’s pH value to increase by up to 12.0. Nevertheless, the pH value drops dramatically by mixing the NaOH-treated sample with LAMP reaction buffer; ultimately, the pH value of NaOH-treated samples was similar to samples without NaOH treatment. Application of magnetic bead methods resulted in purified DNA/RNA thanks to the application of washing steps, and enables the elimination of centrifugation steps necessary in solid-phase extraction methods. Magnetic bead methods are frequently applied in developed POC NAA-based assays. However, these methods have disadvantages too, such as an external magnet source requirement for mixing and their lengthy procedure, which takes from 30 to even 120 min [43,44]. Moreover, Chaudhary et al. [42] revealed that the magnetic bead method resulted in faint LAMP amplification, probably related to Bst polymerase inhibition by magnetic bead particles and ethanol residue.

Our results extend previous studies [45,46,47,48,49,50] by showing that nucleic acid extraction protocols can be simplified without compromising amplification performance. In this study, the QuickExtract protocol was optimised for clinical nasopharyngeal samples by evaluating different incubation temperatures and times. Although the original protocol included two incubation steps (6 min at 65 °C followed by 2 min at 98 °C), our results showed that short high-temperature incubation is sufficient for effective nucleic acid release. No significant differences in Cq values were observed between incubation times of 1–5 min or temperatures ranging from 92 °C to 98 °C. Lowering the temperature during the DNA/RNA extraction step enables the device to operate at reduced temperatures, which improves energy efficiency, enhances safety, and increases the stability of device components, reagents, and sensors.

Several previous studies demonstrated that simplified or extraction-free approaches can be used for pathogen detection [47,51,52]. For example, Fomsgaard et al. showed that simple heat treatment of nasopharyngeal samples could enable direct detection of SARS-CoV-2 RNA without conventional extraction procedures [47]. Similarly, Bruce and colleagues demonstrated that RT-qPCR detection of SARS-CoV-2 can be performed directly from nasopharyngeal swabs without RNA purification [51], while Smyrlaki et al. reported large-scale testing using extraction-free RT-PCR protocols [52]. However, these studies [45,46,47,48,49,50,51,52] typically focused on a single workflow and did not systematically evaluate the influence of incubation temperature, incubation time, or different extraction reagents on nucleic acid recovery and amplification performance. Furthermore, unlike our study, these studies did not verify the applicability of the proposed solutions to various groups of pathogens, whereas in our study, all methods were tested with various Gram-positive and Gram-negative bacterial species and SARS-CoV-2.

An important limitation of this study is that bacterial isolates were derived from laboratory collection cultures rather than primary clinical material. Consequently, inhibitory matrix effects were minimal, which facilitated controlled comparison of extraction efficiency but may not fully reflect the complexity of routine clinical bacterial samples. Nevertheless, even without inhibitors, the differences in efficiency of tested extraction methods were evident.

Our study systematically compared five rapid nucleic acid extraction systems with reference method and multiple incubation regimes, enabling the identification of a simplified one-step protocol that maintains reliable amplification while reducing procedural complexity. Simplifying the extraction workflow is particularly important for point-of-care diagnostics, where rapid sample processing and minimal manual handling are essential. Our results indicate that optimised thermal lysis protocols can maintain reliable amplification while significantly reducing workflow complexity, supporting their integration into “sample-to-answer” POC biosensor platforms. Taken together, these results provide a systematic evaluation of rapid nucleic acid extraction strategies and demonstrate that reliable amplification can be achieved using a simplified one-step thermal lysis protocol.

## 5. Conclusions

Concentrated, pure and high-quality DNA/RNA is the best sample for all NAA methods. To obtain such a perfect sample, a lengthy and multi-stage process needs to be applied, which is not suitable for rapid POC diagnostics. Therefore, the nucleic acid extraction method applied in POC tests needs to result in DNA/RNA purity and quality adequate to selected NAA to maintain high-level test sensitivity and specificity. There is no universal rapid extraction method suitable for all types of samples and all nucleic acid amplification methods. Even the use of quality-controlled commercial kits for rapid nucleic acid extraction does not guarantee the sensitivity and efficacy of the NAA tests applied. In this study, among the five investigated commercial rapid DNA/RNA extraction kits, only QuickExtract has given results comparable to the reference multi-steps method, universally for different samples. However, the speed and simplicity of the extraction could be increased from 8 min, proposed in the two-step protocol by the manufacturer, to even one minute in a modified one-step protocol. Also, the proposed temperature decrease of DNA/RNA extraction step is important in POC device development because it enables the temperature in the device to be lowered, which results in the energy efficiency of the device, increasing the safety and stability of the device elements, reagents and sensors. Nevertheless, the DNA/RNA extraction method has to be carefully selected and validated, especially in terms of types of samples tested and the NAA method used subsequently, as well as the type of investigated microorganism. Inappropriate selection and validation of DNA/RNA extraction methods might lead to false negative results. Even a sample transport medium might have a negative impact on test results. On the other hand, some of the limitations can be minimised by selected NAA methods. Therefore, development of rapid point-of-care (POC) molecular diagnostic tests needs to be validated as an entire process, from sample collection to reading the results.

## Figures and Tables

**Table 1:** Primers used in real-time PCR and RPA.

No	Species of Bacteria	Primer Name	Primer Sequence [5′-3′]	Amplicon Size [bp]	References
1	*Escherichia coli*	lacZ3F	TTGAAAATGGTCTGCTGCTG	234	[5]
		lacZ3R	TATTGGCTTCATCCACCACA		
2	*Klebsiella pneumoniae*	khe-F	TGATTGCATTCGCCACTGG	428	[6]
		khe-R	GGTCAACCCAACGATCCTG		
3	*Shigella dysenteriae*	SdysDF1	TCTCAATAATAGGGAACACAGC	202	[7]
		SdysDR1	CATAAATCACCAGCAAGGTT		
4	*Enterococcus faecium*	EFI_F	ATATCGGCTGTCTCCATGCT	121	[8]
		EFI_R	CCGCCGTCTATAATCCATTC		
5	*Salmonella* Typhimurium	spyF	TTGTTCACTTTTTACCCCTGAA	401	[9]
		spyR	CCCTGACAGCCGTTAGATATT		
6	*Staphylococcus aureus*	Sa-fibF	AATTGCGTCAACAGCAGATGCGAG	210	[10]
		Sa-fibR	GGACGTGCACCATATTCGAATGTACC		
7	*Pseudomonas aeruginosa*	PA431CF	CTGGGTCGAAAGGTGGTTGTTATC	232	[11]
		PA431CR	GCGGCTGGTGCGGCTGAGTC		
8	*Streptococcus pneumoniae*	plyF	GAATTCCCTGTCTTTTCAAAGTC	348	[12]
		plyR	ATTTCTGTAACAGCTACCAACGA		
9	*Corynebacterium diphtheriae*	toxF	ATCCACTTTTAGTGCGAGAACCTTCGTCA	249	[13]
		toxR	GAAAACTTTTCTTCGTACCACGGGACTAA		
10	*Clostridum perfingens*	PL3	AAGTTACCTTTGCTGCATAATCCC	283	[14]
		PL7	ATAGATACTCCATATCATCCTGCT		

**Table 2:** Mean Cq for OFR1ab and gene N for clinical samples extracted using various modification of QuickExtract protocol.

Incubation Time	Incubation Temperature							
	65 °C		92 °C		95 °C		98 °C	
	OFR1ab (SD)	Gene N (SD)	OFR1ab (SD)	Gene N (SD)	OFR1ab (SD)	Gene N (SD)	OFR1ab (SD)	Gene N (SD)
1 min	-	-	28.73 (4.70)	26.71 (4.52)	28.37 (4.48)	26.27 (4.45)	28.66 (4.24)	26.47 (4.27)
2 min	-	-	28.95 (5.04)	26.83 (4.94)	29.07 (4.95)	26.70 (4.65)	28.98 (4.74)	26.64 (4.44)
3 min	-	-	28.47 (4.19)	26.51 (4.06)	29.14 (4.62)	27.01 (4.61)	28.93 (4.58)	26.86 (4.58)
5 min	-	-	29.14 (4.53)	27.21 (4.50)	29.08 (4.43)	26.93 (4.40)	29.09 (4.39)	27.00 (4.31)
10 min	ND	ND	-	-	-	-	-	-

SD—standard deviation (for 3 clinical specimens). ND—not detected.

**Table 3:** Comparison of extraction kits.

Extraction Kit	Speed	Simplicity	Equipment Requirements	Features	Commentary
QuickExtract	~3–11 min *	Streamlined, single-tube procedure, one or two incubation temperature(s)	Vortex, heating block, micropipette, timer	Unpurified nucleic acids, possible presence of amplification step inhibitors	65 °C—negative/unrepeatable results for bacteria, negative results for SARS-CoV-2; for higher temperatures (>90 °C), two-step incubation is not required
ViRNAEx	~5–18 min *	Streamlined, single-tube procedure, one incubation temperature	Vortex, heating block, micropipette, timer	Unpurified nucleic acids, possible presence of amplification step inhibitors	Lower temperature (70 °C vs. 95 °C) works slightly better for Gram-positive bacteria; sample dependent results for SARS-CoV-2
One-Step DNA/RNA Extraction Buffer	~16 min	Streamlined, single-tube procedure, one incubation temperature	Vortex, heating block, micropipette, timer	Unpurified nucleic acids, possible presence of amplification step inhibitors	Negative results for SARS-CoV-2; wide dispersion of results depending on Gram-negative bacterial species; results for Gram-positive bacteria significantly worse than the reference method
Loopamp PURE DNA Extraction Kit	~12 min	Moderately complex procedure, especially for new users, tiring with more samples	Heating block, micropipette, timer	Pre-cleaned DNA sample	Negative results for SARS-CoV-2; forced dilution of the sample, which results in a significant difference compared to the reference method for bacterial samples
Loopamp Viral RNA Extraction Kit	~1 min	One-step, single-tube procedure, no incubation steps	Micropipette	Unpurified nucleic acids, possible presence of amplification step inhibitors	Negative results for SARS-CoV-2
DNeasy Blood & Tissue Kit	~35–105 min **	Highly complex procedure, (especially for Gram-positive bacteria) contains several spinning or vacuuming steps, time consuming incubation(s); demands pipetting and tube changing, tiring with more samples.	Vortex, heating block, centrifuge or vacuum pump, micropipette, timer, demands additive reagents	Pure DNA sample	Reference method
QIAamp Viral RNA Mini Kit	~35 min	Highly complex procedure, contains several spinning or vacuuming steps and room temperature, short incubation, demands pipetting and tube changing, tiring with more samples.	Vortex, centrifuge or vacuum pump, micropipette, timer	Pure RNA sample	Reference method

* depending on incubation time; ** depending on whether the bacteria are Gram-negative or Gram-positive.

**Table 4:** Limit of detection and mean Cq values in real-time PCR for bacterial samples extracted with Loopamp PURE, QuickExtract, and DNeasy Blood & Tissue kits.

	Limit of Detection in cfu/µL (Cq/SD)		
Species of Bacteria	Loopamp PURE DNA Extraction Kit	QuickExtract (95 °C, 5 min)	DNeasy Blood & Tissue Kit
*Escherichia coli*	10.2 (34.87/1.39)	10.2 (33.65/0.88)	10.2 (33.86/0.9)
*Klebsiella pneumoniae*	12.7 (35.71/1.60)	12.7 (33.09/0.86)	1.27 (35.32/0.93)
*Shigella dysenteriae*	98.0 (36.35/0.82)	9.8 (35.95/1.16)	9.8 (35.87/0.97)
*Salmonella* Typhimurium	14.5 (36.30/1.05)	14.5 (33.70/2.03)	14.5 (34.61/1.08)
*Pseudomonas aeruginosa*	14.7 (36.17/1.05)	14.7 (32.99/0.82)	14.7 (33.48/0.73)
*Staphylococcus aureus*	1880 (34.20/1.10)	1880 (33.71/0.61)	18.8 (33.15/0.88)
*Corynebacterium diphtheriae*	430 (34.92/0.48)	43.0 (36.14/1.01)	43.0 (32.87/0.38)
*Enterococcus faecium*	8400 (34.36/0.59)	840 (34.96/0.74)	8.4 (33.24/0.48)
*Clostridum perfringens*	15.8 (35.85/1.25)	15.8 (31.61/0.56)	1.58 (34.46/1.50)
*Streptococcus pneumoniae*	120 (36.20/0.82)	120 (32.72/0.68)	12.0 (33.55/0.38)

SD—standard deviation.

## Data Availability

The original contributions presented in this study are included in the article. Further inquiries can be directed to the corresponding author.
